# Author Correction: A shape-shifting redox foldase contributes to *Proteus mirabilis* copper resistance

**DOI:** 10.1038/s41467-019-08920-9

**Published:** 2019-03-01

**Authors:** Emily J. Furlong, Alvin W. Lo, Fabian Kurth, Lakshmanane Premkumar, Makrina Totsika, Maud E. S. Achard, Maria A. Halili, Begoña Heras, Andrew E. Whitten, Hassanul G. Choudhury, Mark A. Schembri, Jennifer L. Martin

**Affiliations:** 1 Institute for Molecular Bioscience, University of Queensland, St. Lucia, QLD 4072 Australia; 2 School of Chemistry and Molecular Biosciences, University of Queensland, St. Lucia, QLD 4072 Australia; 3 Australian Infectious Diseases Research Centre, University of Queensland, St. Lucia, QLD 4072 Australia; 4 La Trobe Institute for Molecular Science, La Trobe University, Bundoora, VIC 3068 Australia; 5 Griffith Institute for Drug Discovery, Griffith University, Nathan, QLD 4111 Australia; 6 Present Address: Bristol-Myers Squibb, Arnulfstraße 29, 80636 Munich, Germany; 7 Present Address: Department of Microbiology and Immunology, School of Medicine, University of North Carolina, Chapel Hill, NC 27514 USA; 8 Present Address: Institute of Health and Biomedical Innovation, School of Biomedical Sciences, Queensland University of Technology, Kelvin Grove, QLD 4059 Australia; 9 Present Address: School of Human Movement and Nutrition Sciences, University of Queensland, St. Lucia, QLD 4072 Australia; 10 Present Address: Australian Centre for Neutron Scattering, Australian Nuclear Science and Technology Organization, Lucas Heights, New South Wales 2234 Australia; 11 Present Address: Cello Health Consulting, Farnham Surrey, GU9 7DN UK

Correction to: *Nature Communications*; 10.1038/ncomms16065; published online 19 July 2017

This Article contains errors in Fig. 1, Table 1 and the Methods section. In panel c, the labels for PmScsC and EcDsbC in the upper two curves are interchanged. In Table 1 and the Methods section entitled ‘Extended structure’, the space group of the extended PmScsC structure is incorrectly referred to as H3_2_ and should read H32. Correct versions of Fig. 1 and Table 1 are presented below; the errors have not been corrected in the Article.

**Figure 1:**
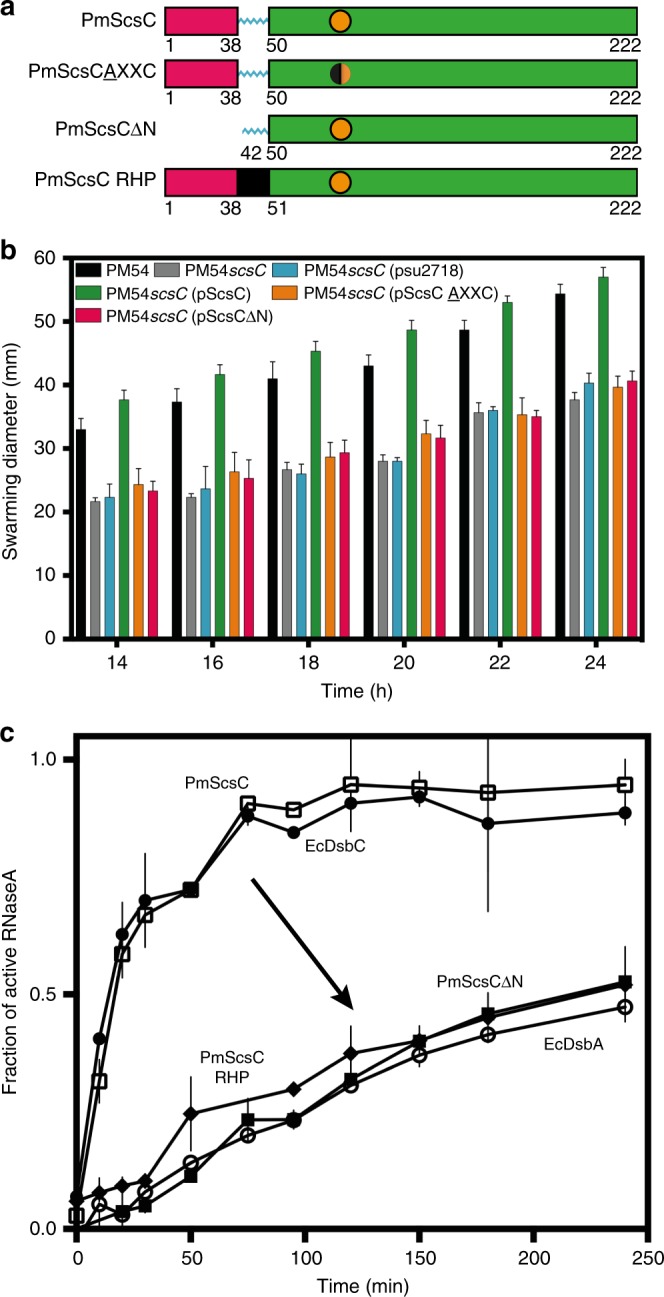
Fig. 1

**Table 1:** PmScsC crystal structure statistics

	Compact (4XVW)	Transitional (5IDR)	Extended (5ID4)
*Data collection*			
Space group	P2_1_	I4	H32
Cell dimensions			
* a*, *b*, *c* (Å)	137.5, 163.9, 181.9	193.1, 193.1, 105.8	86.7, 86.7, 330.9
* α*, *β*, *γ* (º)	90, 90, 90	90, 90, 90	90, 90, 120
Resolution (Å)	91.15–2.60 (2.74–2.60)	136.51–2.56 (2.57–2.56)	110.29–2.92 (2.93–2.92)
* R*_merge_	0.072 (0.617)	0.083 (0.741)	0.059 (0.625)
* I* /*σI*	11.0 (2.0)	14.9 (2.2)	14.2 (2.8)
Completeness (%)	98.6 (95.4)	99.4 (100.0)	99.2 (100.0)
Redundancy	3.8 (3.7)	4.1 (4.1)	4.1 (4.2)
*Refinement*			
Resolution (Å)	91.15–2.60	42.82–2.56	40.36–2.92
No. of reflections	243,409	62,069	10,652
*R*_work_/*R*_free_ (%)	24.8/28.2	17.1/22.2	25.1/26.3
No. of atoms			
Protein	40,850	10,262	1720
Ligand/ion	NA	NA	NA
Water	281	82	0
*B* factors (Å^2^)			
Protein	59.7	50.6	122.2
Ligand/ion	NA	NA	NA
Water	41.5	43.0	NA
RMS deviations			
Bond length (Å)	0.006	0.008	0.010
Bond angles (º)	1.21	1.05	1.17

Single crystals were used to collect each dataset. Values for highest resolution shell are shown in parentheses

