# Supplementary material for: A shape-shifting redox foldase contributes to Proteus mirabilis copper resistance
Source: Nat Commun. 2017 Jul 19;8:16065. doi: 10.1038/ncomms16065 (PMC5524982; doi:10.1038/ncomms16065)
Supplement: Supplementary Information [file ncomms16065-s1.pdf]

File name: Supplementary Information
Description: Supplementary figures, supplementary table and supplementary note.

File name: Supplementary Movie 1
Description: Morphing video of the three crystal structures of PmScsC (4XVW, 5IDR and 5ID4), showing the overall architectural changes in the trimer, synchronised with the conformational changes of the linker peptide. Movies were created by generating morphs between the crystal structures in PyMOL. The morphs were exported as png files and the individual images were assembled into videos using Adobe Premiere Pro. The catalytic thioredoxin fold domains are shown in green, the active sites in orange and the non-catalytic domains in white. One way of detecting the range of motion while watching the video is to track the relative positions of the active sites (orange).

File name: Supplementary Movie 2
Description: Morphing video of the two crystal structures of EcDsbC (1JZD and 1EEJ) showing a rocking motion between the two catalytic domains. Movies were created by generating morphs between the crystal structures in PyMOL. The morphs were exported as png files and the individual images were assembled into videos using Adobe Premiere Pro. The catalytic thioredoxin fold domains are shown in green, the active sites in orange and the non-catalytic domains in white. One way of detecting the range of motion while watching the video is to track the relative positions of the active sites (orange).

File name: Supplementary Movie 3
Description: Morphing video of the two crystal structures of human PDI (4EKZ and 4EL1) showing a larger rotation of one catalytic domain, relative to EcDsbC. Movies were created by generating morphs between the crystal structures in PyMOL. The morphs were exported as png files and the individual images were assembled into videos using Adobe Premiere Pro. The catalytic thioredoxin fold domains are shown in green, the active sites in orange and the non-catalytic domains in white. One way of detecting the range of motion while watching the video is to track the relative positions of the active sites (orange).

File name: Supplementary Movie 4
Description: Morphing video of the three crystal structures of PmScsC (4XVW, 5IDR and 5ID4), showing a much larger range of motion involving twisting and extension of the three catalytic domains. Movies were created by generating morphs between the crystal structures in PyMOL. The morphs were exported as png files and the individual images were assembled into videos using Adobe Premiere Pro. The catalytic thioredoxin fold domains are shown in green, the active sites in orange and the noncatalytic domains in white. One way of detecting the range of motion while watching the video is to track the relative positions of the active sites (orange).

File name: Peer review file
Description:

Supplementary Table 1: SAXS data collection and analysis details

**Data collection parameters**	**PmScsC (*wild type*)**	**PmScsC (*rigid mutant*)**
Instrument	SAXS-WAXS (Australian Synchrotron)	
Beam geometry	Point	
Wavelength (Å)	1.033	
Camera length (m)	1.576	1.485
q-range (Å^{-1})	0.01-0.55	0.01-0.55
Exposure time (s)	28 (14 × 2 s exposures)	23 (23 × 1 s exposures)
Protein concentration (mg/mL)	2.15	1.30
Temperature (°C)	10	10
Normalisation standard	Water	Water
**Structural parameters**		
$I(0)$ (cm^{-1}) [from $p(r)$]	0.1006 ± 0.0002	0.06648 ± 0.00007
R_g (Å) [from $p(r)$]	36.0 ± 0.1	44.1 ± 0.1
$I(0)$ (cm^{-1}) [from Guinier]	0.1010 ± 0.0005	0.0663 ± 0.0001
R_g (Å) [from Guinier]	36.5 ± 0.3	43.8 ± 0.2
D_{max} (Å)	105 ± 5	135 ± 7
Porod volume (Å^3)	97800 ± 9000	108000 ± 10000
R_g (Å) [crystal structures]	30.5 – 35.8	
D_{max} (Å) [crystal structures]	90 – 105	
Dry volume (Å^3) [from sequence]	91000	
**Molecular mass determination**		
Partial specific volume (cm^{3}g^{-1})	0.73	
Contrast, $\Delta\rho$ (10^{10} cm^{-2})	2.75	
Molecular mass M_r [from $I(0)$]*	69900 ± 3500	76400 ± 3500
Molecular mass M_r [from sequence]	74100	73300
**Software employed**		
Primary data reduction	*Scatterbrain*	
Data processing	*PRIMUS and GNOM*	
Rigid body modelling	*Coral and EOM*	

*For the wild type protein, data were also collected at protein concentrations of 4.50 and 8.85 mg/mL. The corresponding $I(0)$ values for these concentrations are 0.2120 and 0.4060 cm^{-1}. These values yield molecular mass estimates of 70.3 and 68.6 kDa. Thus, there is no evidence of significant interparticle interactions in the measured concentration range. For the rigid mutant protein, data were also collected at protein concentrations of 0.60, 2.65 and 5.30 mg/mL. The corresponding $I(0)$ values for these concentrations are 0.0312, 0.1447 and 0.2940 cm^{-1}. These values yield molecular mass estimates of 77.9, 81.6 and 82.9 kDa. Thus, there is evidence of weak interparticle interactions in the measured concentration range that is deemed negligible at the concentration used for data modelling and analysis.

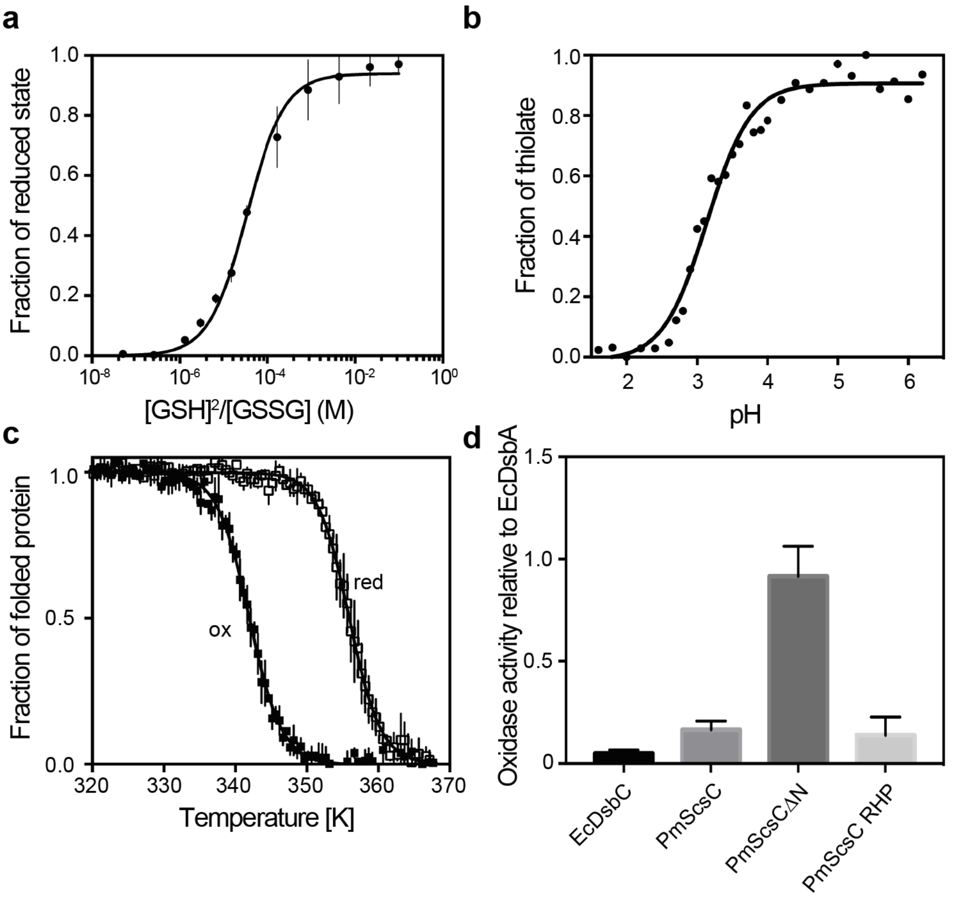

Supplementary Figure 1. Functional properties of PmScsC

a. Redox potential. PmScsC was equilibrated in a range of glutathione (GSSG/GSH) redox buffers to measure the equilibrium constant K_{eq} (34 μM, which corresponds to a redox potential of -108 mV). Data are shown as the mean ± s.d., of three replicate experiments. **b.** Determination of the pK_a of the highly reactive cysteine in the C̲XXC active site motif. Presence of the catalytic thiolate anion is pH-dependent and can be spectroscopically monitored. The pK_a of PmScsC C̲XXC was determined to be 3.1. A representative example of the three replicate experiments is shown **c.** Thermal unfolding of the catalytic core domain PmScsCΔN, shows that its reduced state (T_m^{red} = 358 K) is more stable than its oxidized (T_m^{ox} = 345 K) form. Data are shown as the mean ± s.d., of three replicate experiments. **d.** Rate of dithiol oxidase catalytic activity of 80nM PmScsC, PmScsCΔN and EcDsbC normalized to the activity of the control enzyme EcDsbA. Glutathione was used as the electron donor in the reaction. PmScsCΔN has an activity similar to that of the archetypal dithiol oxidase, EcDsbA, whereas trimeric PmScsC, the PmScsC RHP mutant and dimeric EcDsbC have negligible activity. Data are shown as the mean ± s.d., of three replicate experiments.

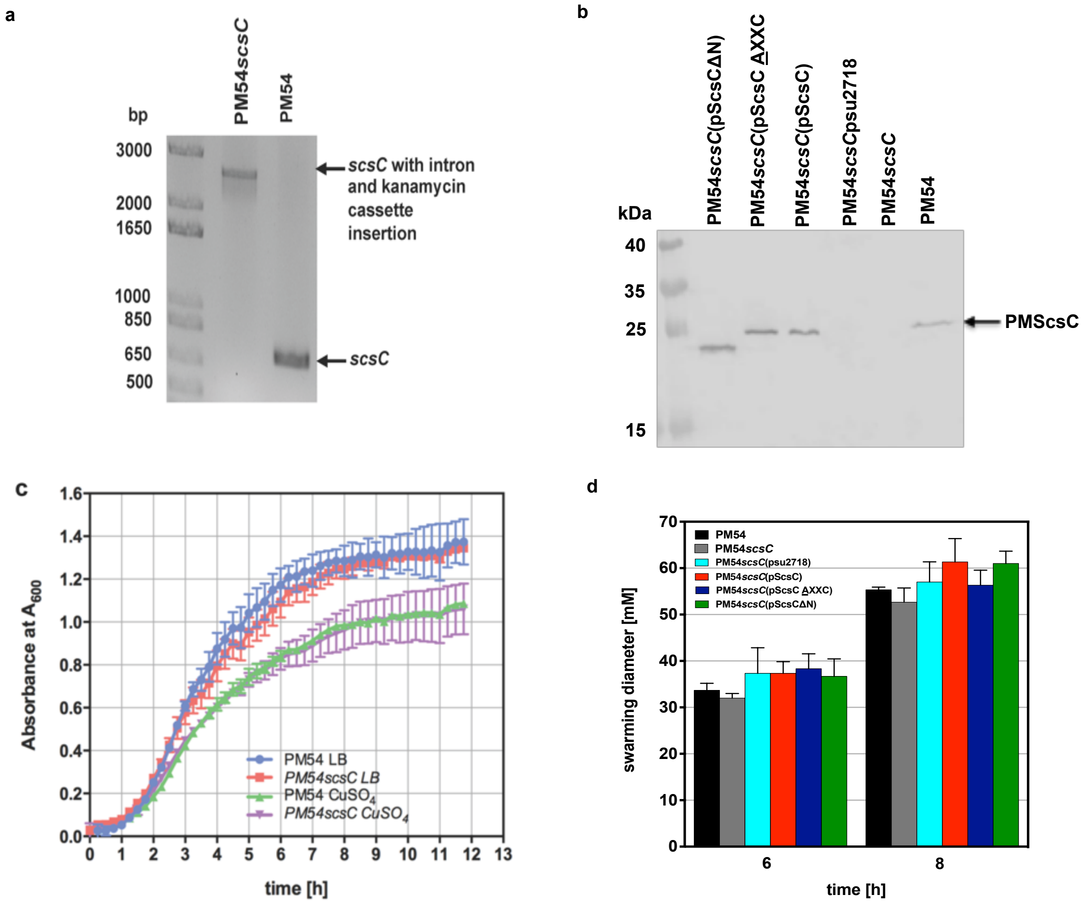

Supplementary Figure 2. Genotypic and phenotypic analysis of PM54 and PM54*scsC*.

a. Agarose gel electrophoresis of *scsC* PCR fragments obtained from PM54 and PM54*scsC*. An amplicon of increased size was obtained from PM54*scsC* (compared to PM54) using primers 5914 and 5915, confirming the correct insertion of the intron and kanamycin cassette in the *scsC* gene. **b.** Western blot analysis of whole cell lysates prepared from PM54, PM54*scsC*, PM54*scsC*(pSU2718), PM54*scsC*(pScsC), PM54*scsC*(pScsC AXXC) and PM54*scsC*(pScsCΔN) using an ScsC-specific polyclonal antiserum. Expression of wild-type ScsC and mutant forms (ScsC AXXC and ScsCΔN) was observed; no expression of ScsC was detected in PM54*scsC*. **c.** Growth analysis of PM54 and PM54*scsC* in LB broth at 37°C in the presence or absence of 1.5 mM CuSO4. There was no significant difference in the growth rate of PM54 (blue) and PM54*scsC* (red) in LB broth, or PM54 (green) and PM54*scsC* (purple) in LB broth containing 1.5 mM $CuSO_4$. Growth was monitored by measuring the optical density at 600nm (OD_{600nm}). Data are shown as the mean ± s.d., of three replicate experiments. **d.** Analysis of PM54 (black), PM54*scsC* (grey), PM54*scsC*(pSU2718) (light blue), PM54*scsC*(pScsC) (red), PM54*scsC*(pScsC AXXC) (dark blue) and PM54*scsC*(pScsCΔN) (green) swarming on LB agar at 37°C at 6 hours and 8 hours post-inoculation. There was no significant difference in the swarming of all strains examined on LB agar in the absence of added $CuSO_4$. Data are shown as the mean ± s.d. deviation, of three replicate experiments.

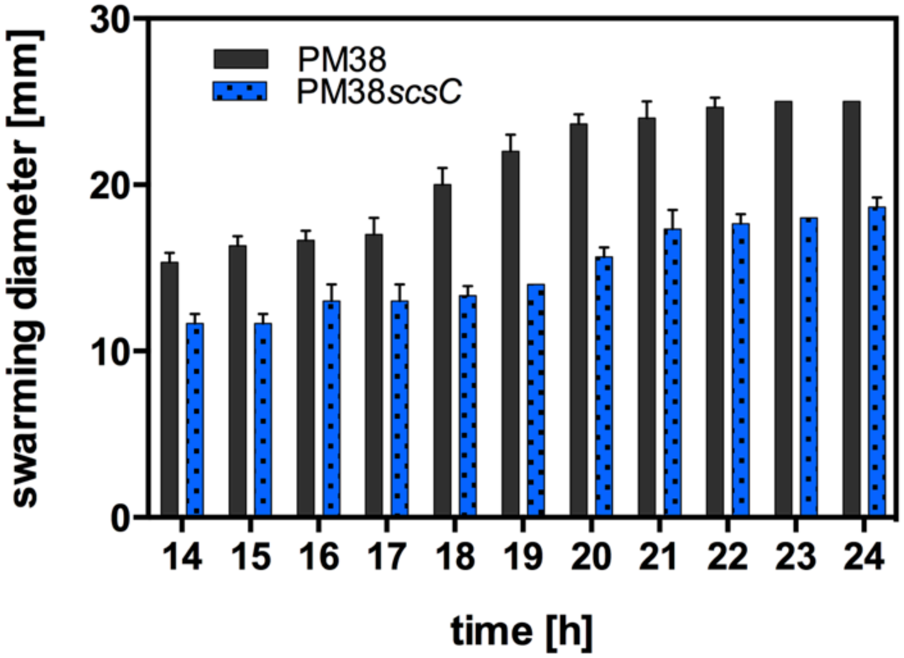

Supplementary Figure 3. Mutant phenotype and function of PmScsC

Difference in swarming motility between wild type PM38 and PM38*scsC* in the presence of 1.5 mM CuSO4 on LB agar plates after incubation for 14 h at 37 °C. PM38*scsC* swarming motility (blue) was significantly reduced compared to wild type PM38 (black; ($P < 0.0001$ for slope calculated by F-test). Data are shown as the mean ± s.d. deviation, of three replicate experiments (in some cases the error bars are too small to be visible).

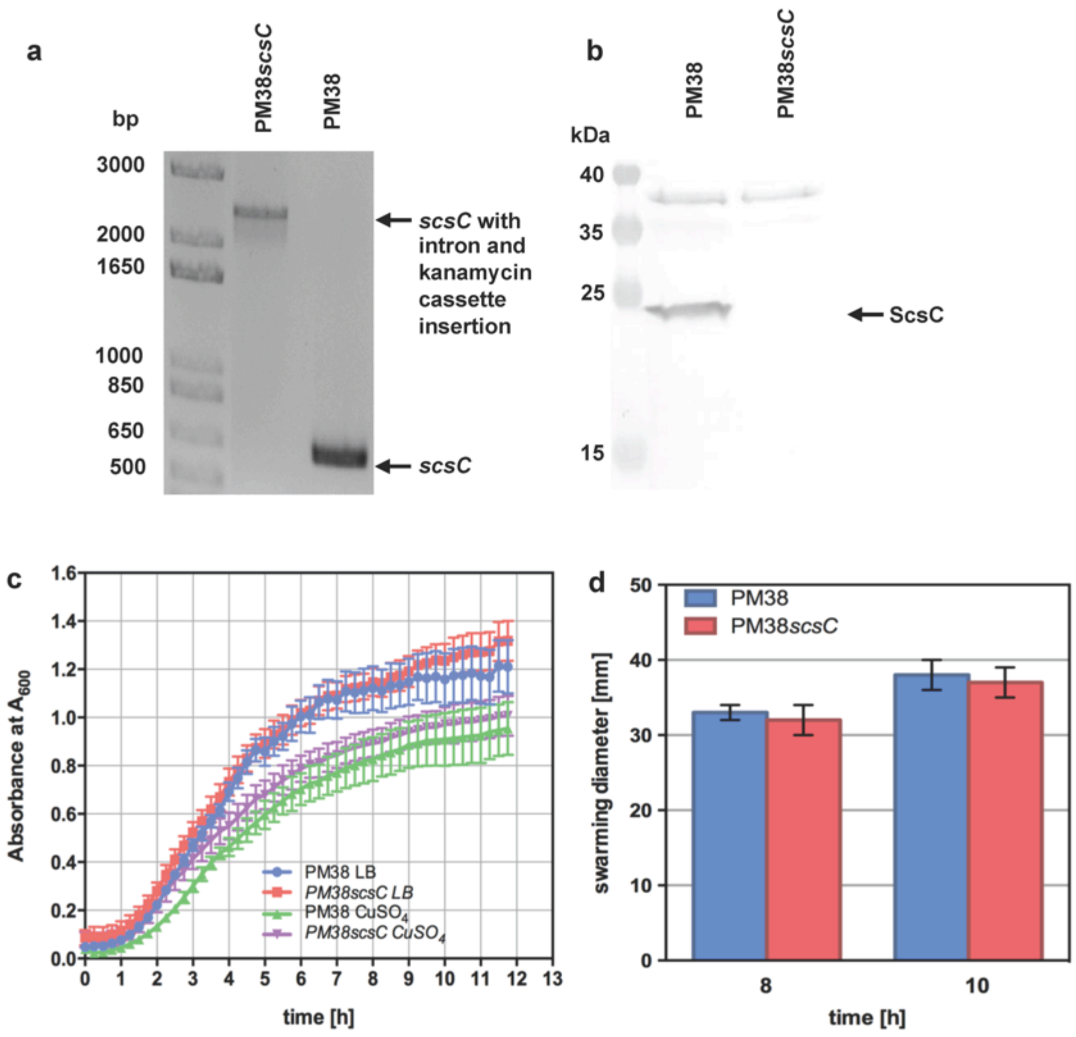

Supplementary Figure 4. Genotypic and phenotypic analysis of PM38 and PM38*scsC*
a. Agarose gel electrophoresis of *scsC* PCR fragments obtained from PM38 and PM38*scsC*. An amplicon of increased size was obtained from PM38*scsC* (compared to PM38) using primers 5914 and 5915, confirming the correct insertion of the intron and kanamycin cassette in the *scsC* gene. **b.** Western blot analysis of whole cell lysates prepared from PM38 and PM38*scsC* using an ScsC-specific polyclonal antiserum. ScsC is expressed by PM38 but not by the PM38*scsC* mutant. **c.** Growth analysis of PM38 and PM38*scsC* in LB broth at 37°C in the presence or absence of 1.5 mM $CuSO_4$. There was no significant difference in the growth rate of PM38 (blue) and PM38*scsC* (red) in LB broth, or PM38 (green) and PM38*scsC* (purple) in LB broth containing 1.5 mM CuSO4. Growth was monitored by measuring the OD_{600nm}. Data are shown as the mean ± s.d., of three replicate experiments. **d.** Analysis of PM38 (blue) and PM38*scsC* (red) swarming on LB agar at 37°C at 8 hours and 10 hours post-inoculation. There was no significant difference in the swarming of PM38 and PM38*scsC* on LB agar in the absence of added $CuSO_4$. Data are shown as the mean ± s.d., of three replicate experiments.

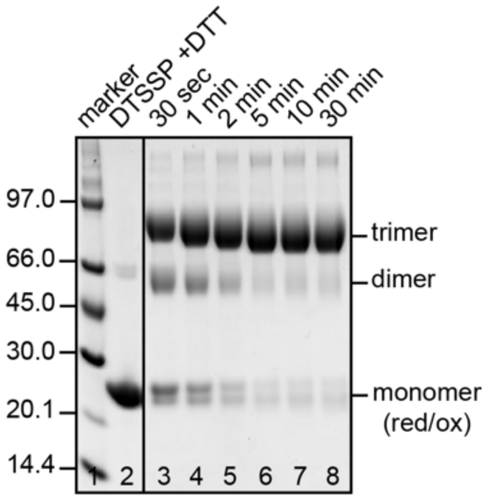

Supplementary Figure 5. SDS gel of cross-linked PmScsC

Denaturing SDS-PAGE (-DTT) of purified PmScsC, incubated for 30 s – 30 min with a disulfide-bonded chemical cross-linker DTSSP. Within 30 s a trimer is evident (lanes 3-8) that can be disrupted by DTT added to the sample after 30 min time (DTSSP + DTT).

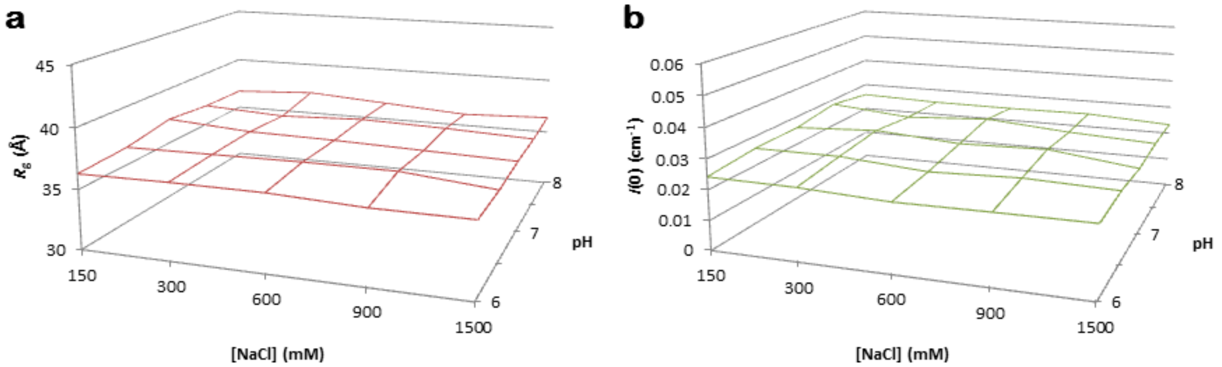

Supplementary Figure 6. PmScsC NaCl/pH gradient scattering data

a. The variation of the radius of gyration is shown as a function of both pH and NaCl concentration. There is no significant or systematic variation with NaCl or pH, indicating that the conformation/flexibility of the protein is not dependent on solution conditions, but is an inherent property of the protein **b.** The variation in the forward scattering, *I*(0), shown as a function of both pH and NaCl concentration. The values are corrected for the change in contrast of the protein in the varying concentration of NaCl. The plot shows the oligomeric state of the protein is independent of both NaCl concentration and pH.

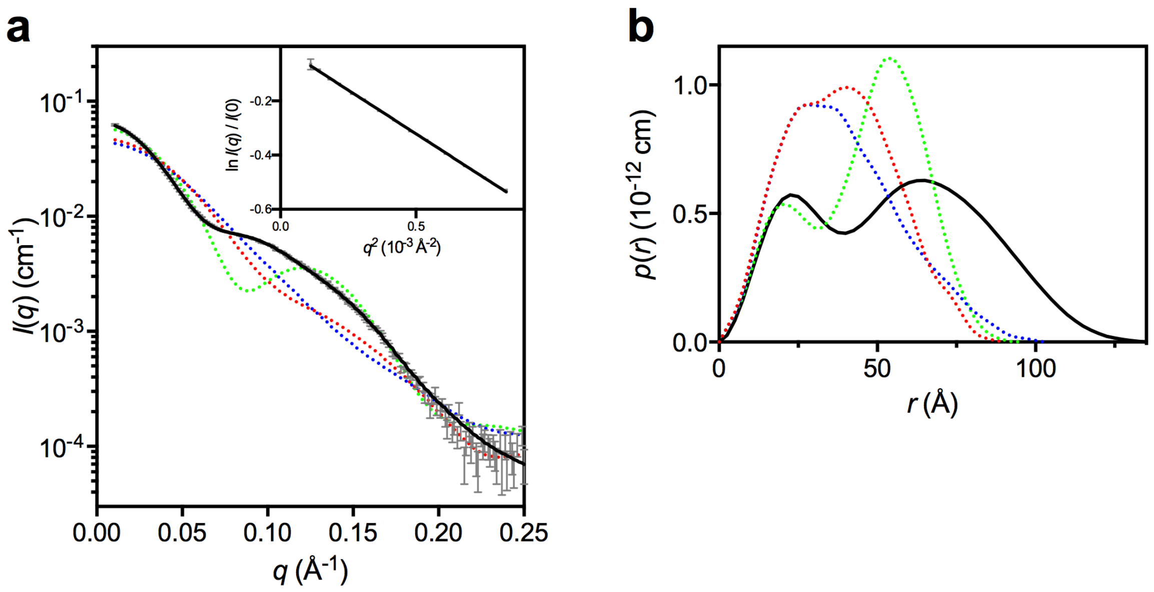

Supplementary Figure 7. PmScsC RHP mutant scattering data

a. Small-angle X-ray scattering data collected from the PmScsC RHP mutant (grey) and the calculated scattering profile of the ensemble model overlayed in black (SASBDB: SASDBW6). The predicted scattering profile of each of the crystal structures is also shown (dashed lines: PDB: 4XVW compact, red; PDB: 5IDR transitional, blue; PDB: 5ID4 extended, green). The agreement between the experimental data and an ensemble model is excellent, yielding χ^2=1.8 (compared to χ^2=1708.1 (compact); χ^2=1710.3 (transitional); χ^2=1365.5). The Guinier region (inset) of the scattering data is linear, consistent with a monodisperse solution. **b.** Pair distance distribution function derived from the scattering data, showing the maximum dimension of the particles in solution is 135 Å. Also shown is the calculated $p(r)$ for each of the other structures (dashed lines: compact, red; transitional, blue; extended, green), showing a maximum dimension of 90, 105, and 100 Å, respectively. The $p(r)$ curves from each of the crystal structures are markedly different to the experimentally determined $p(r)$ curve.

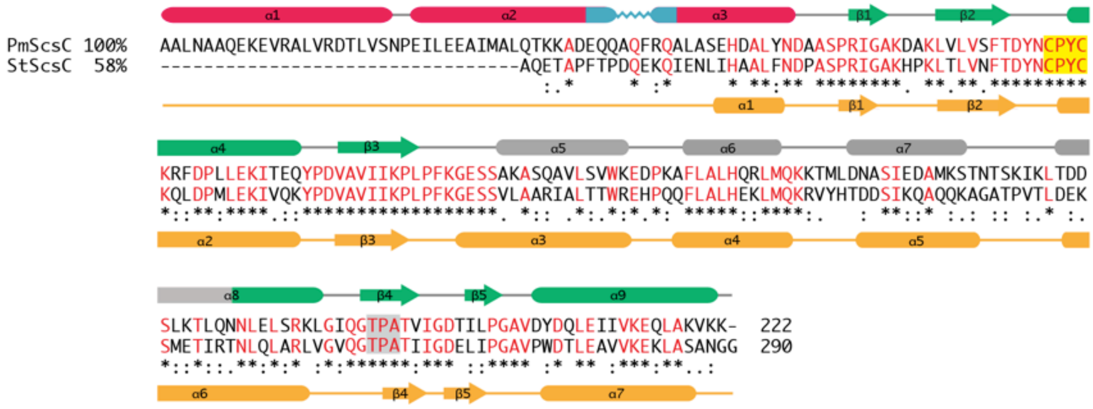

Supplementary Figure 8. Sequence alignment of PmScsC and StScsC

Secondary structure elements are shown for PmScsC (above the amino acid sequence) and StScsC (below the amino acid sequence). Secondary structure elements for PmScsC are colored using the color scheme from Fig. 2a/b, whereas those for StScsC are shown in orange. The CXXC motif is shaded in yellow, and the *cis*Pro motif found in most thioredoxin fold proteins is highlighted in grey.

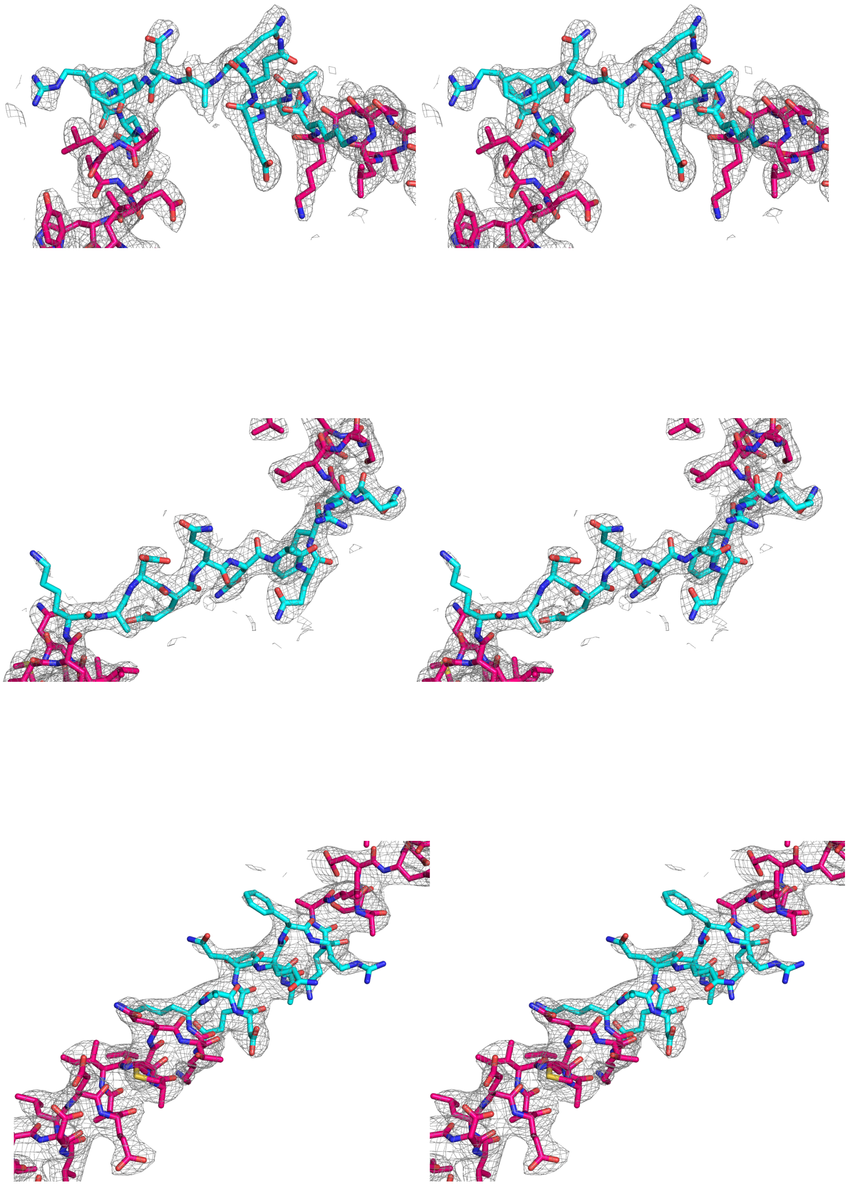

Supplementary Figure 9. Stereo images of representative electron density in the PmScsC crystal structures.

The electron density of the flexible linker region in **a.** compact PmScsC (PDB: 4XVW) **b.** the intermediate protomer of the transitional structure (PDB: 5IDR) and **c.** extended PmScsC (PDB: 5ID4). In each case the linker adopts a different secondary structure, forming **a.** loop, **b.** β-strand or **c.** α-helix. The residues 39-KADEQQAQFRQ-49 are shown in cyan in each case and residues that form the trimerisation stem are coloured magenta. The likelihood-weighted $2F_o - F_c$ electron density maps (contoured to 1σ) are represented by the wire mesh. The maps were generated using phenix.maps and missing F_o were filled with F_c.

Supplementary Note 1: The codon optimized gene sequence used for expression of PmScsC(22-243) is:

```
GCTGCTCTGAATGCTGCCCAAGAAAAAGAAGTGCGTGCACTGGTTCGCGACACCCTGGTCAG
CAACCCGGAAATTCTGGAAGAAGCAATCATGGCTCTGCAGACGAAAAAAGCGGATGAACAGC
AAGCCCAGTTTCGTCAAGCACTGGCTAGCGAACATGATGCCCTGTATAACGACGCAGCATCT
CCGCGCATTGGTGCAAAAGATGCCAAACTGGTGCTGGTTTCTTTTACCGACTATAATTGCCC
GTACTGTAAACGTTTCGATCCGCTGCTGGAAAAAATCACGGAACAGTACCCGGACGTCGCAG
TGATTATCAAACCGCTGCCGTTTAAAGGTGAAAGCTCTGCGAAAGCCTCACAGGCTGTTCTG
TCGGTCTGGAAAGAAGATCCGAAAGCATTCCTGGCTCTGCACCAGCGTCTGATGCAGAAGAA
AACCATGCTGGATAACGCAAGCATTGAAGACGCTATGAAAAGTACCAATACGTCCAAAATCA
AACTGACCGATGACAGTCTGAAAACGCTGCAGAACAATCTGGAACTGTCCCGCAAACTGGGC
ATTCAAGGTACCCCGGCGACGGTTATTGGCGATACCATCCTGCCGGGTGCCGTGGACTATGA
CCAACTGGAAATTATCGTGAAAGAACAACTGGCAAAAGTGAAAAAATGA
```
